# Effectiveness of early hematopoietic stem cell transplantation in preventing neurocognitive decline in aspartylglucosaminuria: A case series

**DOI:** 10.1002/jmd2.12222

**Published:** 2021-05-05

**Authors:** Arthavan Selvanathan, Jane Kinsella, Francesca Moore, Robert Wynn, Simon Jones, Peter J. Shaw, Bridget Wilcken, Kaustuv Bhattacharya

**Affiliations:** ^1^ Genetic Metabolic Disorders Service The Children's Hospital at Westmead Sydney New South Wales Australia; ^2^ Children's Hospital at Westmead Clinical School, the Faculty of Medicine and Health The University of Sydney Sydney New South Wales Australia; ^3^ Manchester Centre for Genomic Medicine University of Manchester Manchester UK; ^4^ NSW Biochemical Genetics Service The Children's Hospital at Westmead Sydney New South Wales Australia; ^5^ Department of Blood and Marrow Transplant Royal Manchester Children's Hospital Manchester UK; ^6^ Blood and Marrow Transplant Service The Children's Hospital at Westmead Sydney New South Wales Australia

**Keywords:** aspartylglucosaminuria, inborn errors of metabolism, lysosomal storage disorders, stem cell transplantation

## Abstract

Aspartylglucosaminuria (AGU) (OMIM #208400) is a recessively inherited disorder of glycoprotein catabolism, a subset of the lysosomal storage disorders (LSDs). Deficiency of the enzyme *glycosylasparaginase* (E.C. 3.5.1.26) leads to accumulation of aspartylglucosamine in various organs and its excretion in the urine. The disease is characterized by an initial period of normal development in infancy, a plateau in childhood, and subsequent regression in adolescence and adulthood. No curative treatments are currently available, leading to a protracted period of significant disability prior to early death. Hematopoietic stem cell transplantation (HSCT) has demonstrated efficacy in other LSDs, by providing enzyme replacement therapy in somatic viscera and decreasing substrate accumulation. Moreover, donor‐derived monocytes cross the blood‐brain barrier, differentiate into microglia, and secrete enzyme in the central nervous system (CNS). This has been shown to improve neurocognitive outcomes in other LSDs. The evidence to date for HSCT in AGU is varied, with marked improvement in glycosylasparaginase enzyme activity in the CNS in mice models, but varying neurocognitive outcomes in humans. We present a case series of four children with AGU who underwent HSCT at different ages (9 years, 5 years, 5 months, and 7 months of age), with long‐term follow‐up post‐transplant (over 10 years). These cases demonstrate similar neurodevelopmental heterogeneity based on formal developmental assessments. The third case, transplanted prior to the onset of neurocognitive involvement, is developing normally despite a severe phenotype in other family members. This suggests that further research should examine the role of early HSCT in management of AGU.

**Synopsis**

Hematopoietic stem cell transplantation in Aspartylglucosaminuria prior to neurocognitive decline may result in an attenuated phenotype.

## 1 INTRODUCTION

Aspartylglucosaminuria (AGU) (OMIM #208400) is a recessively inherited disorder of glycoprotein degradation, with increased prevalence in the Finnish population.^1^ Deficiency of the enzyme glycosylasparaginase (E.C. 3.5.1.26), which cleaves the link between protein and carbohydrate moieties of glycoproteins, causes accumulation of aspartylglucosamine, which is subsequently excreted in the urine.^2^ Clinically, the disorder is characterized by a period of normal growth until age 2, a plateau phase of slowed development through childhood, and rapid deterioration in early adulthood.^3^ Despite this, median life expectancy is around 45 years of age, suggesting a prolonged period of poor neurocognitive function and limited quality of life prior to death. Currently, as no curative treatments exist, therapy is limited to symptomatic management of disease manifestations, such as antiepileptic medications for seizures.^4^

Because of the lack of enzyme replacement therapy (ERT) available for AGU, development of treatments which target both visceral and neurocognitive manifestations is crucial. Given the success of hematopoietic stem cell transplantation (HSCT) in other storage disorders, such as Mucopolysaccharidosis Type I (MPS I) (OMIM #607014),^5^ it merits consideration as a potential therapy in AGU. Multipotent stem cells are able to differentiate into the tissues of various organs and provide enzyme replacement at these sites. In particular, some donor‐derived monocytes cross the blood‐brain barrier and differentiate into microglia that are capable of secreting glycosylasparaginase.^6^ Theoretically, HSCT could therefore reduce build‐up of aspartylglucosamine in the central nervous system (CNS).

Evidence for treatment of AGU with HSCT is sparse. We present a case series of four individuals with AGU who underwent HSCT at a range of ages (5 months, 7 months, 5 years, and 9 years) to add to the available literature.

## 2 METHODS

Cases of AGU with early evidence of neurocognitive involvement, who presented to The Children's Hospital at Westmead from 2000 onwards, were offered HSCT. Informed consent was obtained from all three families for retrospective review of their medical records, as well as publication. A single case from the Royal Manchester Children's Hospital has also been included in the case series. Ethics approval was provided by the Sydney Children's Hospital Network Research Ethics and Governance Team (Human Research Ethics Committee Reference Number: CCR2019/27).

## 3 RESULTS

### 3.1 Case 1

#### 3.1.1 Presentation

**FIGURE 1:**
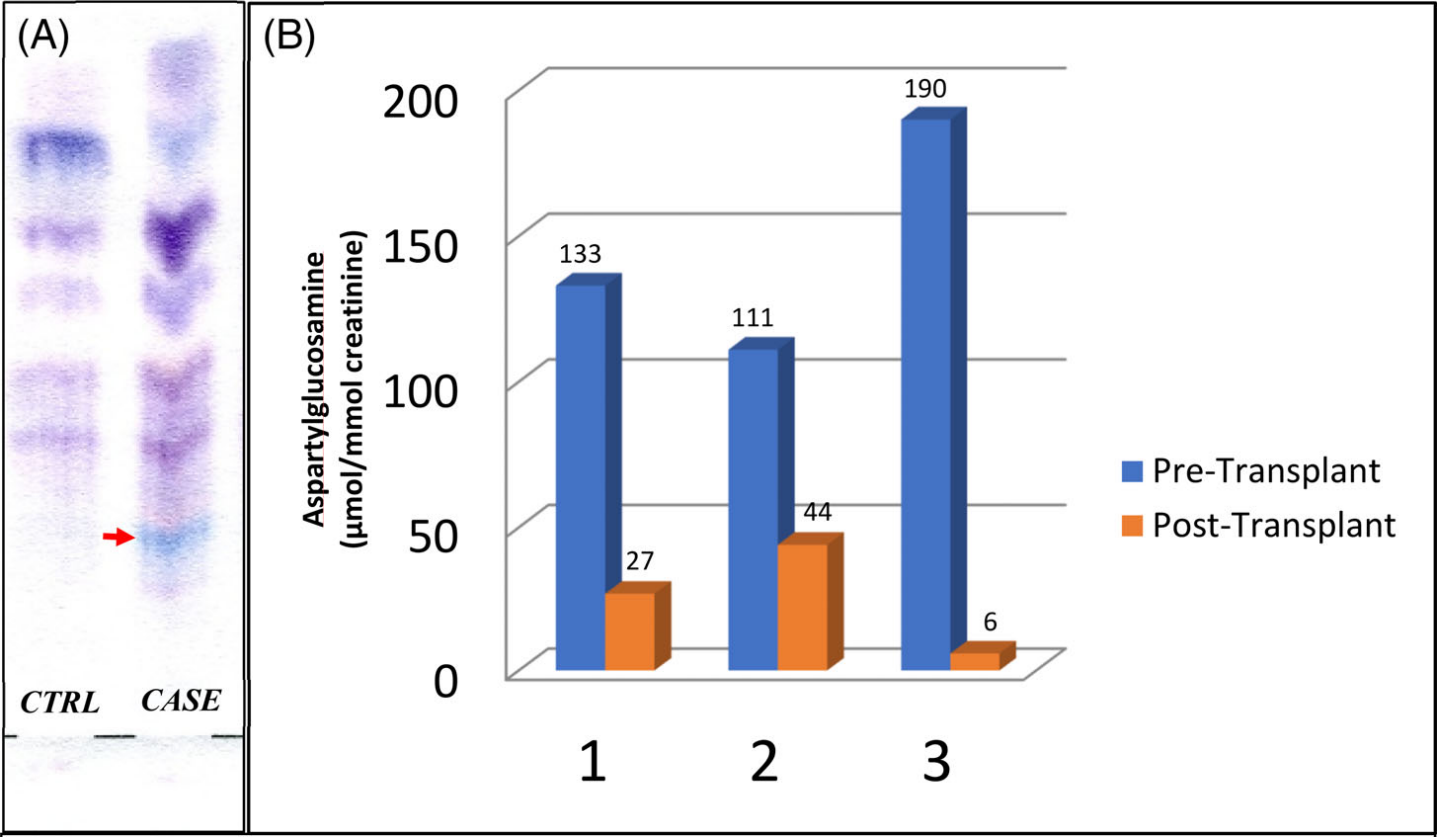
A, Presence of a band corresponding to aspartylglucosamine (arrow) in a pre‐transplant sample from case 3, analyzed by high‐voltage electrophoresis chromatography, with corresponding absence in the control sample. B, Urinary aspartylglucosamine excretion quantified by liquid chromatography tandem mass spectrometry in three patients pre‐ and post‐transplant, demonstrating improvement in cases 1 and 2 and normalization in case 3

Case 1, a girl currently aged 19 years, presented initially as an 8 years old with toe‐walking, increasing clumsiness, and developmental delay (Table 1). There was an initial period of normal motor development, though speech was always delayed. She had recurrent otitis media as a child and recurrent pre‐septal cellulitis secondary to a tooth abscess. On presentation, she had characteristic coarse facial features, flat feet, mild hepatomegaly, and poor concentration. Urine amino acid analysis via liquid chromatography tandem mass spectrometry demonstrated increased levels of aspartylglucosamine (Figure 1), and diagnosis was confirmed with low activity of glycosylasparaginase in leukocytes (Table 1).

**TABLE 1:** Baseline data before transplant for four patients (A) with clinical and biochemical outcomes (B)

(A) Baseline data before transplant				
Patient	1	2	3	4
Presenting features	Coarse facial features, toe walking, increased clumsiness	Expressive/receptive speech delay, poor socialization	Detected by newborn familial screening	Detected by newborn familial screening
Age at diagnosis	8 years 7 months	4 years 8 months	9 days	5 days
Aspartylglucosaminidase activity	Leukocytes: 0.4 nmol/24 h/prot (28‐35)	Leukocytes: 4.7 nmol/24 h/mg prot (118)	Leukocytes: 3.2 pmol/min/mg prot (48‐49)	Leukocytes: 0.19 nmoL/mL/h prot (10‐60)
Genotype	‐	c.167C>T (homozygous) p.(A56V)	c.794G>A, c.940+1G>A p.(R265H), p.(?)	‐
Predicted effect on AGA	‐	Absent from controls, and the majority of in silico tools predict it to be pathogenic	c.794G>A: absent in controls, in silico tools: pathogenic c.940+1G>A: canonical splice variant	‐
Height (centile)	52nd	50th	35th	25th
Weight (centile)	85th	93rd	1st	9th
Head circumference	—	75th	—	2nd
MRI brain	Normal	Pineal cyst, structurally normal	—	Normal
Age at transplant	9 years 3 months	5 years 4 months	5 months	7 months

(B) Post‐transplant Assessments				
Age at last follow‐up	19 years	12 years	12 years	11 years
Last developmental assessment	7 years post HSCT: moderate intellectual disability particularly in the area of comprehension	3 years post HSCT: moderate intellectual disability, decreased processing speed verbal memory attention span	7 years post HSCT: normal development—mild psychomotor slowing and difficulties sustaining attention	10 years post HSCT: skills remain substantially behind the majority of peers in all areas assessed
Height (centile)	32nd	36th	43rd	25th
Weight (centile)	96th	98th	10th	>99th
Head circumference (centile)	60th	50th	15th	—
MRI brain	Mildly increased cerebellar atrophy	—	Stable left frontal lesion (6 mm × 5 mm), likely glioneuroma	Age appropriate scan with no specific abnormality
Ongoing issues	Primary ovarian failure, moderate intellectual disability, poor attention, kyphosis, aggressive	Obesity, moderate intellectual disability requiring some help with ADLs	Epilepsy (resolved, now off medications)	Obesity, psoriasis, moderate intellectual disability, and bilateral hearing loss poor attention
School	Support unit with personalized learning plan	Mainstream school with support teacher	Mainstream school with support teacher	Special educational needs schooling;personalized support
Aspartylglucosaminidase activity	Leukocytes: 23 nmol/24 h/mg protein (28–35)	Leukocytes: 21.8 nmol/24 h/mg protein (28‐44)	Leukocytes: 36 nmol/min/mg protein (50)	Plasma: 2.2 nmol/mL/h (10‐60) Leukocytes: 20.2 nmol/mg/h (18.1)

She had a neurocognitive assessment pre‐transplant (at 9 years of age) using the Wechsler Intelligence Scale for Children, Fourth Edition (WISC‐IV). This demonstrated moderate intellectual impairment (developmental age of 6 years), with particularly poor comprehension and attention (at the level expected of a 3 years old).

#### 3.1.2 Transplantation

Case 1 received a 6/6 HLA‐matched sibling donor bone marrow transplant at 9 years of age, using myeloablative conditioning with busulfan and cyclophosphamide. Her post‐transplant course included infections while neutropenic (*Bacillus* species and coagulase‐negative staphylococcus), which responded well to antibiotics, and acute graft‐versus‐host disease of the skin which subsequently improved.

#### 3.1.3 Outcome

Case 1 is now 10 years post‐transplant. She had a moderate reduction in aspartylglucosamine (Figure 1). She was assessed post‐transplant using the Stanford‐Binet Intelligence Scales, Fifth Edition (SB5), which demonstrated moderate intellectual disability (Table 2). She speaks in full sentences and can help with cleaning around the house. A cousin in Jordan is of a similar age and also affected with AGU: this cousin has not received disease‐modifying treatment. He achieved similar developmental milestones but has regressed, and is now nonverbal.

**TABLE 2:** Pre‐ and post‐transplant developmental assessments for four patients with aspartylglucosaminuria

Patient	1		2		3		4	
	*WISC‐IV (at 9 years of age)*		*DAS‐II (at 5 years of age)*		No assessment performed pre‐transplant		No assessment performed pre‐transplant	
Pre‐transplant	Moderate intellectual disability (estimated <0.1st centile), particularly in the areas of comprehension, attention, and executive functioning: standard scores and centiles not available.		Verbal IQ	3rd centile				
			Nonverbal IQ	13th centile				
			Spatial IQ	0.1st centile				
			*Full‐scale IQ*	*1st centile*				
			*ABAS‐II (at 5 years of age)*					
			Conceptual	1st centile				
			Social	5th centile				
			Practical	6th centile				
			*Global assessment*					
			*Composite*	*2nd centile*				
Post‐transplant	*SB5 (at 16 years of age)*		*SB5 (at 14 years of age)*		*WISC‐IV (at 7 years of age)*		*WISC‐IV*	
	Nonverbal IQ	<0.1st centile	Non‐verbal IQ	<0.1st centile	Verbal comprehension	55th centile	Verbal comprehension	0.3rd centile
	Verbal IQ	<0.1st centile	Verbal IQ	<0.1st centile	Perceptual reasoning	14th centile	Perceptual reasoning	0.1st centile
	*Full‐scale IQ*	*<0.1st centile*	*Full‐scale IQ*	*<0.1st centile*	Working memory	9th centile	Working memory	0.1st centile
					Processing speed	4th centile	Processing speed	4th centile
					*Full‐scale IQ*	*13th centile*	*Full‐scale IQ*	*0.1st centile*
	*Vineland‐3 (at 14 years of age)*		*Vineland‐3 (at 14 years of age)*		*ABAS‐II (at 7 years of age)*			
	Communication	<1st centile	Communication	Centile	Conceptual	58th centile		
	Daily living	<1st centile	Daily living	Centile	Social	50th centile		
	Socialization	<1st centile	Socialization	3rd centile	Practical	—		
	*Adaptive behaviour*		*Adaptive behaviour*		*Global assessment*			
	*Composite*	*<1st centile*	*Composite*	*1st centile*	*Composite*	*55th centile*		

*Note*: For IQ scores, 50th percentile is the mean, 5th percentile is −2 SD, and 95th percentile is +2 SD from mean.

There are additional ongoing issues with primary ovarian failure, poor attention, and aggressive behaviors at home. Nonetheless, she now has employment in a supermarket and catches the bus to work under the supervision of a support worker.

### 3.2 Case 2

#### 3.2.1 Presentation

Case 2, a boy, initially presented at 5 years of age with language delay and poor socialization. His early development was normal; he sat at 5 months, said his first words at 9 months, and walked at 11 months. He required bilateral grommet insertion due to recurrent otitis media. A formal developmental assessment at 5 years of age using the Differential Ability Scales, Second Edition (DAS‐II) noted delays in nonverbal reasoning (13th centile), language comprehension and expression (third centile), and fine motor skills (first centile).

Urine amino acid analysis demonstrated gross increase in aspartylglucosamine; leukocyte glycosylasparaginase activity was significantly reduced (Table 1).

#### 3.2.2 Transplantation

Case 2 received a 4/6 HLA‐matched sibling donor bone marrow transplant at 5 years and 4 months of age, with busulfan and cyclophosphamide conditioning. He was discharged 2 months post‐transplant on a weaning course of steroids for gastrointestinal graft‐versus‐host disease. Aside from adenoviral infection (subsequently treated), he had no further transplant‐related complications.

#### 3.2.3 Outcome

Two years post‐transplant, the glycosylasparaginase level for case 2 improved, albeit still below the reference range; correspondingly the aspartylglucosamine excretion improved but was still elevated (Figure 1). His last assessment, using the SB5 at age 14 years, demonstrated a stable moderate intellectual disability (Table 2). He is nonetheless in year 6 at school with a personalized learning plan and support teacher, and has maintained long‐term friendships with children from his kindergarten class.

### 3.3 Case 3

#### 3.3.1 Presentation

Case 3, a boy, was detected in the neonatal period, as he was born into an Iranian Mandean family with extensive consanguinity and known cases of AGU. A urine sample collected on the first day of life demonstrated grossly elevated aspartylglucosamine, and leukocyte glycosylasparaginase activity was reduced (Table 1). Despite the consanguinity, genetic studies revealed compound heterozygous variants in *AGA*.

#### 3.3.2 Transplantation

Case 3 received a 5/6 HLA‐matched unrelated donor cord blood transplant at 5 months of age. He was conditioned with busulfan and cyclophosphamide. The post‐transplant course included stage III graft‐versus‐host disease, which was managed effectively with steroids. Other subsequent complications included left supraclavicular venous thrombosis (treated with heparin and warfarin) and intermittent hemolytic anemia thought to be secondary to minor antigen incompatibility (treated with further steroids). Both of these have resolved.

#### 3.3.3 Outcome

Case 3 has done well post‐transplant. Biochemically he has normal enzyme function, with normal glycosylasparaginase activity and aspartylglucosamine excretion (Table 1 and Figure 1). His weight initially tracked below the third centile, but since age 8 has stayed between the 10th and 50th centiles. He had a period of intermittent seizures at age 8 and was commenced on sodium valproate, but this was ceased at age 9 with no further recurrences. There was an incidental finding of a glioneuroma in the left frontal area, which has not increased in size on subsequent imaging. He has some issues with anxiety, for which he receives cognitive behavioral psychotherapy.

From a neurocognitive standpoint, his developmental assessment 7 years post‐transplant showed “average” verbal comprehension, adaptive functioning, and executive functioning, with relative weaknesses in processing speed and attention (Table 2). He is now in year 9 at school, with learning support for mathematics and English.

### 3.4 Case 4

#### 3.4.1 Presentation

Case 4 was detected in the neonatal period with blood and urine samples collected on the day one of life. The family, of Kashmiri origin, have four older children, three of whom are affected by AGU and two who also have Fabry's disease. Urine amino acid chromatography showed a significant band corresponding to aspartylglucosamine (not quantified) and plasma glycosylasparaginase activity was decreased (Table 1).

#### 3.4.2 Transplantation

Case 4 received a 6/6 HLA‐matched unrelated donor cord blood transplant at 7 months of age. She received IV busulfan and cyclophosphamide conditioning and anti‐thymocyte globulin (ATG) for serotherapy. Post‐transplant complications included grade 1 graft‐versus‐host disease of the skin, managed with topical and parenteral steroids. Case 4 had evidence of veno‐occlusive disease, despite low busulfan levels. This was managed supportively and recovered without further intervention.

#### 3.4.3 Outcome

The most recent follow‐up for this patient took place 10 years post‐transplant; she has remained clinically well. She has moderate bilateral hearing impairment requiring hearing aids and is obese, being screened for complications and supported by a dietitian.

Case 4 shows biochemical stability. Urine chromatography was still able to detect a band corresponding to aspartylglucosamine; however, this was reduced compared to pre‐transplant. In leukocytes, plasma glycosylasparaginase activity was comparable to controls.

Neurocognitive assessment 10 years following transplant was undertaken using the Wechsler Intelligence Scale for Children, Fourth Edition (WISC‐IV), which showed a moderate intellectual disability (Table 2). She remains substantially behind her peers and continues in a special education school; however, her family report improvement in language and there is no evidence of regression. Her function is also better in comparison to affected brothers who did not receive stem cell transplantation, with less challenging behavior.

## 4 DISCUSSION

### 4.1 Diagnosis of AGU

Patients with AGU often come to medical attention due to an early growth spurt and development of macrocephaly, speech delay, and clumsiness.^3^ Recurrent respiratory infections are also common and may precede neurocognitive delay.^7^

Diagnosis is confirmed either by analyzing glycosylasparaginase enzyme activity or by genetic testing for mutations within the highly conserved AGA gene. The condition has much higher prevalence in Finland^1^: over 95% of Finnish cases result from homozygosity for one specific mutation, c.488 G➔C.^8^

It was recognized in the early 2000s that a significant number of non‐Finnish cases of AGU were also present, often in consanguineous families and due to different variants. There are some known mutations in particular populations^9^; however, genotype‐phenotype correlations are yet to be robustly determined.

There is currently also limited data on newborn screening for AGU across the world. Mononen et al^10^ used a fluorometric glycosylasparaginase assay to test 6564 cord blood samples and detected one additional case of AGU in an apparently normal term baby girl, where there was no preceding family history. However, given the perceived lack of evidence‐based therapies to create benefit from early detection, there has not been further evaluation of newborn screening for AGU.

### 4.2 Treatment options for AGU

Management of AGU is currently symptomatic and non‐curative. Seizures occur in 2% of children and 28% of adults and are treated with antiepileptics.^11^ Umbilical and inguinal hernias are treated with herniorraphy.^7^

ERT has shown promise in multiple other lysosomal storage disorders (LSDs), particularly in reducing build‐up of accumulated substrate in tissues other than the CNS.^12^ ERT has been trialed for AGU using synthetic glycosylasparaginase, in a previously validated mouse model of the disease.^13^ There was widespread reduction in aspartylglucosamine in visceral organs following synthetic glycosylasparaginase administration, but only 20% reduction in brain tissue; earlier treatment was associated with a greater reduction in brain aspartylglucosamine.^14^ However, no studies have translated into trials with human participants.^3^ ERT also comes with other drawbacks, including the inconvenience of weekly infusions, alloantibody production, and the financial cost of lifelong treatment, which is in the order of over $200 000 per year per patient.^15^ Similar to other enzyme replacement therapies, neurocognitive decline is unlikely to be ameliorated.

### 4.3 The rationale and evidence for HSCT in AGU

Bone marrow transplantation was first trialed as a treatment for a LSD by Hobbs et al^16^ in a patient with MPS I. They noted evidence of biochemical correction, improvement in visceral manifestations, and arrest of neurocognitive decline. With increasing clinical experience, robust selection criteria for transplantation have been developed for patients with MPS I, including lack of significant pre‐existing neurocognitive impairment.^17^ The mechanism of neurocognitive stabilization is thought to be due to donor‐derived monocytes crossing the blood‐brain barrier, differentiating into microglia and secreting deficient enzyme within the CNS.^6^

HSCT is also considered standard of care in Mucopolysaccharidosis Type VII (MPS VII; OMIM #253220), alpha‐mannosidosis (OMIM #248500), and X‐linked adrenoleukodystrophy (OMIM #300100),^5^ with increasing evidence for success in others such as Mucopolysaccharidosis Type II (OMIM #309900) if completed prior to neurocognitive decline.^18^

The role of HSCT in AGU is less certain. Laine et al^19^ trialed HSCT in 8‐week‐old mice. This resulted in detectable enzyme levels in the liver and spleen with associated reduction in cytoplasmic vacuolization; however, there was persistent AGU and no effects on the CNS. Early HSCT in mice (at three weeks of age) led to detectable glycosylasparaginase activity and reduction in neuronal cell vacuolation.^20^

Evidence for HSCT in humans with AGU is primarily from small case series. Three patients with the major Finnish mutation underwent HSCT; the first two of these patients (both with carrier family members as donors) had successful engraftment but no long‐term neurocognitive data were reported (^21^).

Malm et al^22^ reported on the progress of two patients more than 5 years post‐HSCT, who were initially part of a five‐case series with 14 control non‐transplanted AGU patients.^23^ Both showed radiological evidence of neurocognitive stabilization, yet had more significant neurocognitive deterioration than the controls, with seizures and behavioral disturbances. Arvio et al concluded that transplantation beyond infancy for AGU could not be endorsed.

### 4.4 Our experience with HSCT in AGU

Our four cases add to the limited literature involving HSCT in AGU and provide a similarly heterogeneous set of outcomes. All cases showed good biochemical evidence of engraftment, with reduction in urinary aspartylglucosamine excretion as demonstrated in Figure 1, as well as improved glycosylasparaginase enzyme activity in leukocytes. Plasma enzyme activity was still low post‐transplant when measured (in case 4) given the contribution to this value of nontransplanted tissues. Cases 1 and 2 both had evidence of developmental delay prior to HSCT and still need some assistance in order to function independently. They have now reached adulthood without losing skills, which is not in keeping with the previously reported natural history.^3^

Cases 3 and 4, on the other hand, underwent HSCT prior to significant neurocognitive delay, because of early diagnosis due to previously affected family members. Case 3's development falls into the borderline range: this is unexpected for children with AGU, who would normally have moderate intellectual impairment at this age. However, case 4 also received early HSCT but has a moderate intellectual disability, though she is doing relatively well compared to her affected brothers. It is possible that earlier diagnosis by newborn screening or other similar modalities in high‐risk populations is required in order for HSCT to be maximally beneficial. This is especially important considering the current absence of any other disease‐modifying therapy. However, given case 4's clinical trajectory, it may be that other factors, such as genotype and intensity of allied health therapies, also play a role in modulating the outcome.

Our cases also demonstrate toxicities related to HSCT. Transplant‐related mortality at 1 year for children with inborn errors of metabolism undergoing HSCT in Australia and New Zealand between 2009 and 2019 is 13.2%, averaged across different donor types (I. Nivison‐Smith, Australasian Bone Marrow Transplant Recipient Registry, personal communication, September 24, 2020). This is improved from previous estimates, such as the 58% 3 year survival from 1994 to 2004 worldwide data in MPS I post‐transplant.^24^ Morbidity is also considerable (Table 1), and long‐term consequences such as infertility are also important to consider.^25^ Improving the patient's clinical condition prior to transplant can help improve outcomes.^26^

## 5 CONCLUSION

AGU is a rare glycoprotein storage disorder, but one that causes considerable morbidity through childhood, adolescence, and adulthood. There are currently no disease‐modifying therapies that have been shown to improve survival and quality of life. HSCT theoretically achieves replacement of the deficient enzyme in both the somatic viscera and the CNS. Our case series demonstrates the heterogeneous outcomes seen with HSCT in AGU, but does suggest that transplantation prior to neurocognitive decline may result in an attenuated phenotype. Further evaluation of this in larger case series across countries, as well as the development of standardized tools to assess response to therapy, will be crucial in developing an evidence‐based treatment strategy. Additionally, consideration of newborn screening for AGU, especially in countries such as Finland with higher incidence, is important in order to facilitate early diagnosis and improve clinical outcome.

## CONFLICT OF INTEREST

The authors declare no conflict of interest.
